# A Case of Spontaneous Multiple Abdominal Wall Hematomas in a COVID-19 Patient

**DOI:** 10.7759/cureus.29647

**Published:** 2022-09-27

**Authors:** Mohammad N Alzoubi, Ikram Salameh, Awni D Shahait, Asma Al-zyoud, Soulat Raza

**Affiliations:** 1 General Surgery, The University of Jordan, Amman, JOR; 2 Surgery, Wayne State University School of Medicine, Detroit, USA; 3 Anesthesia and Intensive Care, Al-Istiklal Hospital, Amman, JOR; 4 General Surgery, Queen Elizabeth Hospital Birmingham, Birmingham, GBR

**Keywords:** acquired coagulopathy, conservative management, covid-19, abdominal wall hematoma, spontaneous hemorrhage

## Abstract

Despite being relatively uncommon, abdominal wall hematomas can occur due to blunt trauma, post-percutaneous procedures, anticoagulation, and even spontaneous bleeding. It can present with varying symptoms from acute abdominal pain to life-threatening bleeding causing hypovolemia and shock. With the coronavirus disease 2019 (COVID-19) pandemic, affected patients developed coagulopathy putting patients at risk of venous thromboembolism or excessive bleeding. Herein, we report a case of spontaneous multiple abdominal wall hematomas in a patient after a COVID-19 infection, which was managed conservatively.

## Introduction

Abdominal wall hematoma is a relatively uncommon diagnosis in patients with blunt abdominal trauma. The most common risk factor of abdominal wall hematoma is coagulopathy. It is usually self-limiting and treated with conservative management in stable patients whereas expanding hematoma in hemodynamically unstable patients may need surgical or angiographic treatment [1].

The reported literature suggests that rectus sheath hematoma is associated with blunt abdominal trauma, especially in those on anticoagulation therapy as other abdominal wall hematomas [2]. Spontaneous cases without associated trauma and anticoagulation are more infrequently reported [3].

The predisposing factors for spontaneous rectus sheath hematoma are excessive coughing, pregnancy, and female gender in their 50s [3]. Spontaneous cases can be challenging to diagnose as it mimics common abdominal conditions, such as acute appendicitis, diverticulitis, and intra-abdominal mass.

In the current global context of coronavirus disease 2019 (COVID-19), we report a case of rectus sheath hematoma in a patient with COVID-19 infection. Various extrapulmonary complications and organ dysfunction have been reported with COVID-19 which includes renal, liver, and neurological complications as the most common ones. Platelet dysfunction resulting in thrombocytopenia is commonly observed in patients with COVID-19 [3]. The result is endothelial damage resulting in macro and microvascular thrombosis, and endothelium damage. Hemorrhagic complications are rarer. So rectus sheath hematoma in COVID-19 patients should be kept in mind as a serious vascular complication.

## Case presentation

A 58-year-old woman presented to the emergency department with abdominal pain that had started three days earlier. She had a past medical history of poorly controlled hypertension, diabetes mellitus type 2, and hemorrhagic stroke nine years ago with no residual weakness. She also reported having a COVID-19 infection a week prior to this presentation which required two days of hospitalization in another hospital from which the patient had self-discharged against medical advice.

The abdominal pain was increasing in severity and was aggravated by the dry cough that she already had due to COVID-19. There were no reported red flag symptoms of changes in bowel habits, bleeding per rectum, loss of appetite, or weight loss. The abdominal pain was associated with decreased urine output, general malaise, and dizziness.

On examination, her hemodynamic parameters were normal except for tachycardia, indicating grade II shock. Her BMI was 24.6 kg/m^2^ but she appeared pale and generally unwell. Abdominal examination revealed a right-sided abdominal wall mass with overlying bruising and tenderness but no signs to suggest peritonism. Routine laboratory investigations showed hemoglobin of 4 gm/dL, white cell count of 36.0 × 10^9^/L, and acute kidney injury (AKI) with a creatinine that had risen from a baseline of 1.5 mg/dL to 4.9 mg/dL. Her clotting profile including prothrombin, international normalized ratio (INR), and bleeding times was normal.

The patient was treated and resuscitated in line with care of critically ill surgical patients (CCrISP) protocol [4]. Immediate resuscitation was initiated with crystalloid followed by transfusion of blood products in a 1:1:1 ratio. A total of four units of blood were transfused during her hospital stay, and prophylactic use of broad-spectrum antibiotics in view of severe inflammatory response syndrome (SIRS) response and raised white cell count. The patient was kept under intensive care monitoring for three days.

**Figure 1:**
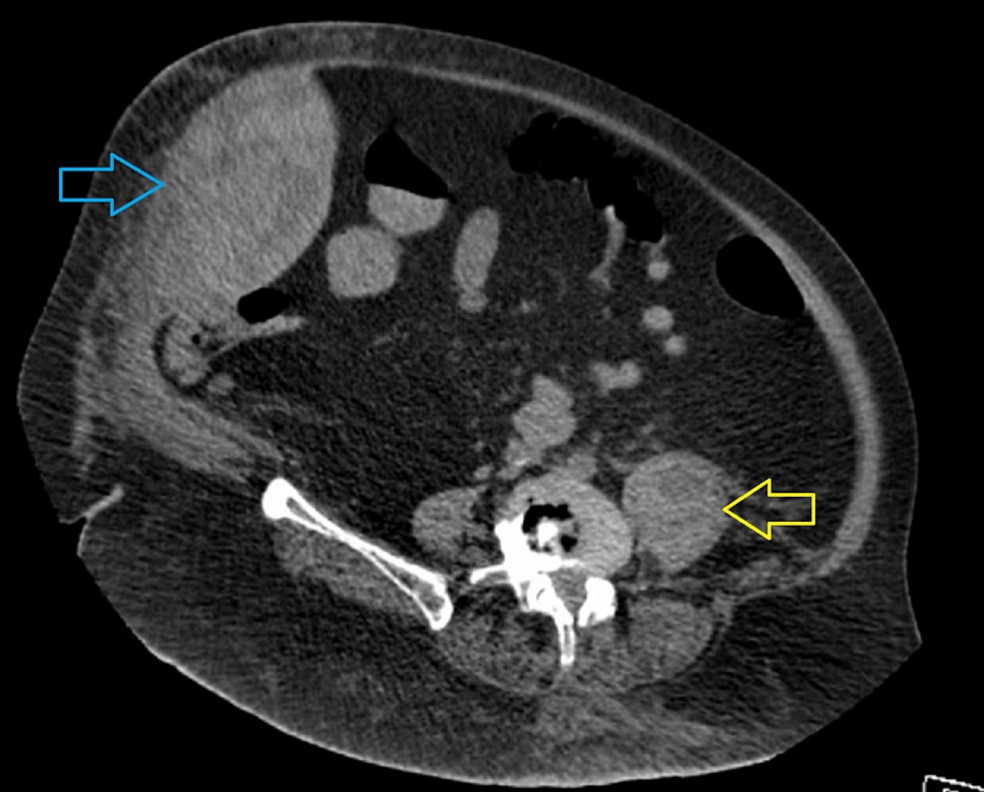
Right rectus sheath (blue arrow) and left iliopsoas (yellow arrow) hematomas on CT scan.

**Figure 2:**
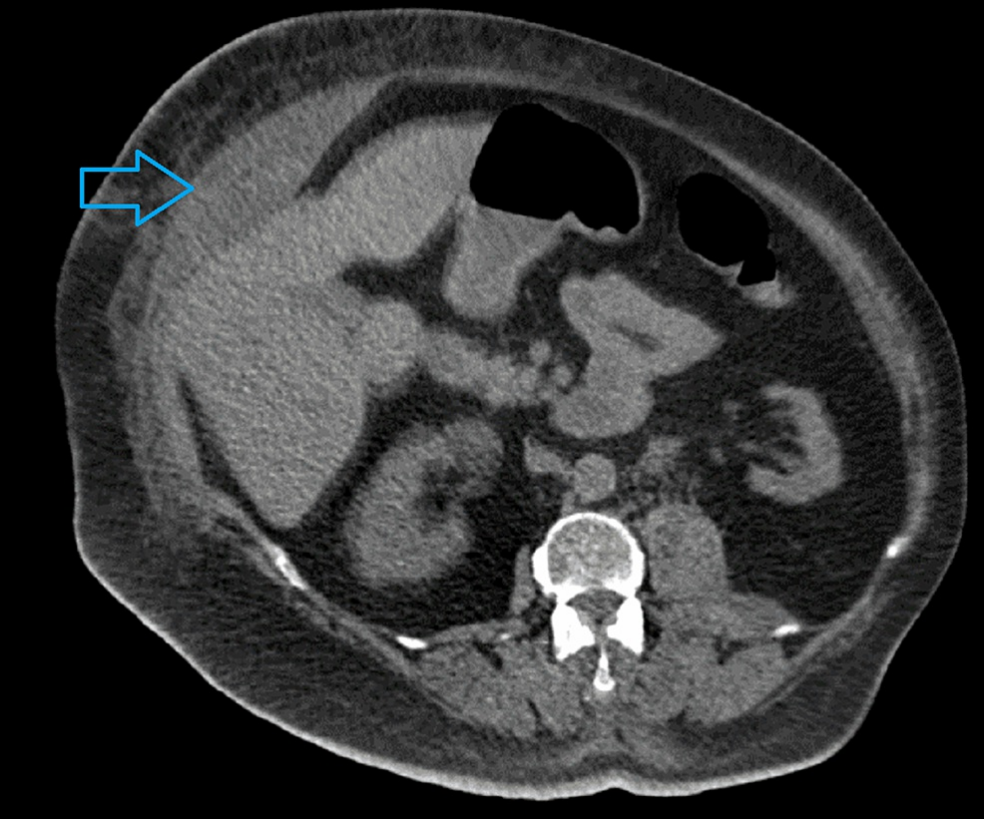
Right internal oblique hematoma (arrow) on the same CT scan.

Once the patient was appropriately resuscitated, we decided to proceed with a non-contrast abdomen and pelvis computed tomography (CT) scan in view of AKI. The CT revealed a 22 x 14 x 12 cm right rectus sheath and internal oblique hematoma extending to the symphysis pubis (Figures 1, 2). Adjacent fatty stranding and a smaller left intramuscular iliopsoas hematoma was also noted.

In view of her current COVID-19 infection, a hematological consultation was sought, and further extensive hematological workup including screening for bleeding disorders and disseminated intravascular coagulopathy (DIC) screen came back negative.

The patient’s hemodynamic parameters stabilized and her urine output improved with resuscitation, clinically there were no signs to suggest active ongoing bleeding, therefore we decided to treat her conservatively. After three days of conservative management, the patient improved further with normalization of hemoglobin reaching 11 gm/dL and return of kidney functions to baseline level. 

On day 5, she was discharged home and saw the clinic again two weeks later with a follow-up CT scan which showed that the hematomas appeared stable and more liquified.

Hereafter the patient was followed up for nearly 6 weeks at regular intervals (every two weeks) and made a complete recovery without requiring radiological drainage or surgical intervention for the hematoma.

## Discussion

Rectus sheath hematoma (RSH) is an uncommon condition, spontaneous rectus sheath hematoma is even more infrequent. They can present with a variety of clinical symptoms and signs from a slow insidious bleed to a rarely life-threatening emergency. The predisposing factors for RSH include blunt abdominal trauma, female gender, elderly age group, anticoagulation therapy, pregnancy, and persistent cough [5]. The more prevalent and widespread use of anticoagulation therapy is believed to be the main reason behind increased incidence and reporting of rectus sheath hematoma in recent literature [6].

In our case, the patient had two of the documented predisposing factors - female gender and persistent cough for a week. Additionally, we believe that the COVID-19 virus itself may have increased her tendency to bleed by predisposing her to functional thrombocytopenia which was not detected in routine testing. Sharifi-Razavi et al. reported the case of a male patient with no significant comorbidities, affected by COVID-19, at the time of presentation, the patient had altered mental status along with fever and cough. An extensive workup was performed and the patient was found to have a massive intracranial hemorrhage [7]. The suggested theory was that brain angiotensin-converting enzyme-2 (ACE2) could be involved in COVID-19 infection pathophysiology and it could lead to alteration of the vascular autoregulatory mechanisms causing the bleed [7].

Two cases of iliopsoas hematomas developed in patients infected with the COVID-19 virus were reported recently. Both patients developed interstitial pneumonia and were supported with non-invasive ventilation [8]. Moreover, a case of a neck and upper torso spontaneous hematoma in a patient on low molecular weight heparin (LMWH) as chemical prophylaxis [9].

Moreover, Rogani et al. reported cases of spontaneous bleeding in patients with COVID-19 infection. All of these patients were on a therapeutic dosage of LMWH - a case of ruptured superior thoracic artery complicated by left pectoral muscle hematoma; another patient with left iliopsoas muscle hematoma; a third patient with large RSH due to both bilateral inferior epigastric arteries rupture and lastly a large left thigh hematoma. Even though all of them were on anticoagulation, the authors suggested that the temporal sequence suggests that COVID-19 was a contributing factor in the development of spontaneous muscle hematoma [10].

## Conclusions

COVID-19 infection is associated with coagulopathy that increases the risk of both thromboembolism, as well as bleeding in affected patients. We reported a unique case of a patient infected with COVID-19 who had not received any anticoagulation therapy and presented with multiple abdominal wall hematomas. The patient was resuscitated and managed conservatively, without any morbidities. A high index of suspicion of RSH should be maintained in patients who present with abdominal pain and mass along with COVID-19 infection, especially in elderly patients even in the absence of anticoagulation therapy or trauma.
